# Population pharmacokinetic modeling and dosing simulation of avalglucosidase alfa for selecting alternative dosing regimen in pediatric patients with late-onset pompe disease

**DOI:** 10.1007/s10928-023-09874-8

**Published:** 2023-08-03

**Authors:** Gilles Tiraboschi, David Marchionni, Gilles Tuffal, David Fabre, Jean-Marie Martinez, Kristina An Haack, Patrick Miossec, Barbara Kittner, Nadia Daba, Fabrice Hurbin

**Affiliations:** 1 Pharmacokinetics Dynamics and Metabolism, Translational Medecine & Early Development, Sanofi R&D, 371 Rue du Pr Blayac, Montpellier, 34184 France; 2 Sanofi Chilly-Mazarin, 1 Avenue Pierre Brossolette, Chilly-Mazarin, 91385 France; 3 Global Pharmacovigilance, Sanofi, Bridgewater, NJ 08876 USA; 4 Global Medical Affairs, Sanofi Gulf Level 3, One JLT, Jumeirah Lake Towers, PO Box 53899, Dubai, UAE

**Keywords:** Alternative dosing, Bodyweight, Enzyme replacement therapy, Pediatric LOPD, PopPK, Pompe disease

## Abstract

Avalglucosidase alfa (AVAL) was approved in the United States (2021) for patients with late-onset Pompe disease (LOPD), aged ≥ 1 year. In the present study, pharmacokinetic (PK) simulations were conducted to propose alternative dosing regimens for pediatric LOPD patients based on a bodyweight cut-off. Population PK (PopPK) analysis was performed using nonlinear mixed effect modeling approach on pooled data from three clinical trials with LOPD patients, and a phase 2 study (NCT03019406) with infantile-onset Pompe disease (IOPD: 1–12 years) patients. A total of 2257 concentration-time points from 91 patients (LOPD, n = 75; IOPD, n = 16) were included in the analysis. The model was bodyweight dependent allometric scaling with time varying bodyweight included on clearance and distribution volume. Simulations were performed for two dosing regimens (20 mg/kg or 40 mg/kg) with different bodyweight cut-off (25, 30, 35 and 40 kg) by generating virtual pediatric (1–17 years) and adult patients. Corresponding simulated individual exposures (maximal concentration, C_max_ and area under the curve in the 2-week dosing interval, AUC_2W_), and distributions were calculated. It was found that dosing of 40 mg/kg and 20 mg/kg in pediatric patients < 30 kg and ≥ 30 kg, respectively, achieved similar AVAL exposure (based on AUC_2W_) to adult patients receiving 20 mg/kg. PK simulations conducted on the basis of this model provided supporting data for the currently approved US labelling for dosing adapted bodyweight in LOPD patients ≥ 1 year by USFDA.

**Supplementary Information:**

The online version contains supplementary material available at 10.1007/s10928-023-09874-8.

## Introduction

Pompe disease is a rare autosomal recessive lysosomal glycogen storage disorder (GSD type II),[1] causing generalized tissue lysosomal glycogen accumulation especially in skeletal, cardiac, and smooth muscles [2]. The incidence of Pompe disease varies by ethnicity [3] with a combined prevalence estimated to be 1 in 40,000 affecting males and females equally [4]. Based on the residual acid alpha-glucosidase (GAA) activity, this disease can be categorized as infantile-onset type (IOPD; GAA activity: <1%) and late-onset type (LOPD; GAA activity: 2−40%) [4]. Majority of the GAA gene mutations are private and occur in compound heterozygosity, hence establishing an accurate correlation between a particular GAA mutation and the clinical presentation or progression of Pompe disease is challenging [5]. In general, residual GAA activity is inversely proportional to disease severity, higher activity is correlated with better prognosis [6, 7].

Alglucosidase alfa was the first approved (2006) enzyme replacement therapy (ERT) using recombinant human GAA in patients aged ≥ 8 years with LOPD [8, 9]. The effectiveness of alglucosidase alfa was limited by poor targeting to skeletal muscles, low affinity for cation-independent mannose 6-phosphate (M6P) receptor (CI-MPR) leading to reduced muscle uptake, and production of humoral immune response interfering with its clinical efficacy [10, 11]. Avalglucosidase alfa (AVAL) is a next-generation recombinant human α-glucosidase (neo-rhGAA) with greater affinity for the M6P receptors [11]. The COMET study (NCT02782741) provided evidence of clinically meaningful improvements in adult patients with LOPD (only one adolescent) treated with AVAL (20 mg/kg every 2 weeks[q2w]) over alglucosidase alfa in respiratory function, functional endurance, motor function, ambulation, and health-related quality of life [12].

There has been a paucity of data for pediatric LOPD patients owing to the challenges in recruiting pediatric patients. However, the Food and Drug Administration (FDA) and International Council for Harmonisation (ICH) have illustrated various approaches to interpolate or extrapolate from the existing adult data or data in pediatric patients in other age groups, or both [13–15]. As per the FDA pediatric study planning and extrapolation algorithm, the present study was based on the ‘complete extrapolation of efficacy’ approach for determining the appropriate dose for pediatric LOPD patients [13, 14].

Notably, there is a continuum of disease spectra between IOPD and LOPD observed from the data in the Pompe registry, indicating that the LOPD phenotype in young children overlaps with IOPD, whereas older children may show similar clinical manifestations as adults [4, 16]. In addition, the clinical program demonstrated safety of AVAL in adult and pediatric patients with LOPD or IOPD (age range: 1–78 years) with a positive benefit-risk over the broad spectrum of Pompe disease [12, 17, 19, 20]. Thus, it can be proposed that the effect of AVAL is likely to be similar between IOPD and LOPD patients, thereby supporting the extrapolation of data from adult to pediatric LOPD patients.

The mini-COMET study (NCT03019406) has been conducted on pediatric patients (aged 1–12 years) with IOPD receiving AVAL (20 and 40 mg/kg q2w) [17]. Therefore, the present analysis utilized the mini-COMET data and combined both type of patients (adult and pediatric) in a population pharmacokinetic (PopPK) model. This proposed model was used to simulate AVAL exposure parameters in adult and pediatric patients and select the appropriate bodyweight dosing adjustment in pediatric patients with LOPD. This study is an extension to a previous PopPK analysis on LOPD adult patients that included only one LOPD adolescent patient [18].

## Methods

### Study population

This PopPK analysis was based on data from three clinical trials (NCT01898364 [Phase 1][19], NCT02032524 [Phase 1/2][20] and NCT02782741 [Phase 3]) [12] in adolescent (only 1) and adult patients with LOPD with an addition of an open label, phase 2 trial (NCT03019406) in pediatric patients with IOPD (< 18 years), showing clinical decline and suboptimal clinical response to alglucosidase alfa (Table 1). In the phase 2 trial (25 week), patients received ascending doses of either intravenous (IV) AVAL (20 mg/kg [n = 6]; 40 mg/kg [n = 10]) q2w, or previously received stable dose of alglucosidase alfa (20 mg/kg q2w to 40 mg/kg every week [n = 6]). The four clinical studies included in the PopPK analysis are described in terms of size, dose, duration, and sampling timepoints in Table 1. All studies were conducted in accordance with Good Clinical Practice (GCP) guidelines and the ethical principles that have their origin in the Declaration of Helsinki. The protocols were approved by the ethics committee or institutional review board at each site, and all participants provided written informed consent.

**Table 1:** Studies included in the population pharmacokinetic analysis

Study	N^a^	Description	Dose and duration	Sampling timepoints
NCT01898364	24	Phase 1, open-label study in treatment naïve and alglucosidase alfa treated LOPD patients	5, 10 and 20 mg/kg q2w for 24 Wks	• Pre-dose, EOI then 1, 2, 4, 8, 12, 16, 24, 32 and 48 h after EOI on Wk 1,13 and 25
NCT02032524	19^b,c^	Phase 1/2, open-label study in LOPD patients	5^d^, 10^d^ or 20 mg/kg q2w for 6 years	• Pre-dose, EOI, 1, 4, 8, 12, and 24 h after EOI on Wk 26, Wk 52 and then once a year
NCT02782741	51	Phase 3, randomized, double-blind study in treatment naïve LOPD patients	20 mg/kg q2w for 49 Wks	• Pre-dose, EOI, 2, 4, 6 and 8 h after EOI on Wk 1 and Wk 49 • Pre-dose and 2 h after the EOI on Wks 13, 25 and 37
NCT03019406	16	Phase 2, randomized, open-label study in alglucosidase alfa treated IOPD patients	20 and 40 mg/kg q2w for 25 Wks	• Pre-dose, EOI, 2, 4, 6 and 8 h after EOI on Wk 1 and Wk 25 • Pre-dose and 2 h after EOI on Wk 13 • For patients treated with 40 mg/kg, additional samples at 12 and 16 h after EOI are planned.

EOI, end of infusion; h, hours; IOPD, infantile-onset Pompe disease; LOPD, late-onset Pompe disease; q2w, once in every 2 weeks; Wk, week

^a^For patients included in NCT02782741 and NCT03019406, only patients receiving avalglucosidase alfa treatment are considered; ^b^One patient had no PK data; ^c^These 19 patients are continuing from NCT01898364 in this follow-up study; ^d^Patients receiving 5 or 10 mg/kg q2w progressively shifted to 20 mg/kg q2w

### Bioanalysis

The plasma concentrations of free AVAL were quantified by using validated fluorometric enzyme activity assay as described previously [18]. The assay was validated between 0.012 and 0.0125 (lower limit of quantitation, LLOQ) and 3.0 µg/mL. The antidrug-antibody (ADA) assay methods were developed according to the current standards for ADA investigation with the implementation of a three-tiered approach, i.e., a first testing method, a confirmatory assay and a final assay to determine ADA titers [18].

### Population pharmacokinetic analysis

#### Software

The PopPK analysis was performed using a nonlinear mixed effect modelling (NONMEM) software (version 7.4.1, Icon Development Solutions, Ellicott City, MD, USA) [21]. Statistical and graphical outputs were generated using the R programming and statistical language (version 3.6.3).

#### Model development, allometric scaling, and covariate screening

Model parameters were estimated using the first-order conditional estimation (FOCE) algorithm with interaction option as implemented in NONMEM [21]. Total PopPK dataset was obtained by supplementing the LOPD (adolescent and adult) dataset described in previous analysis [18] by IOPD (pediatric) dataset. The pharmacostatistical model (PSM) developed in the previous study [18] was used as an initial model for the present analysis by re-optimizing the parameters. The concentration versus time curves were described by a concatenated three-compartment model, with drug back redistribution from the third compartment to the central compartment. The elimination process was characterized by linear and non-linear kinetics in the central compartment. Log-normal inter-individual variability of the parameters was used.

Considering that the dataset included both adult and pediatric patients, time-varying bodyweight dependent allometric scaling of the PSM was tested before the covariate selection process by including the weight on clearance [CL] and distribution volume(s) [V] parameters using following equation:

$$TV(parameter) = \theta (parameter) \times {\left( {\frac{{WT}}{{MedianWT}}} \right)^{\theta (x)}}$$

A sensitivity analysis was performed by optimizing or fixing the allometric scaling factor parameter θ(x) ([CL] = 0.75 and [V] = 1).

Potential PK covariates were then selected based on physiological and clinical relevance and included: age (years), gender, bodyweight (kg), renal function (measured by creatinine clearance normalized by body size or surface area, CL_CRN_), albumin (g/L), alanine amino transferase (IU/L), aspartate amino transferase (IU/L), alkaline phosphatase (IU/L), total bilirubin (µmol/L) and creatine kinase (IU/L). The baseline information was used for each covariate except for age and bodyweight for which both baseline and time varying values were considered. The occurrence of ADA, the disease type (LOPD versus IOPD) as well as previous treatment with alglucosidase alfa status were also investigated. Potential covariates were examined using stepwise selection, including the forward inclusion (alpha risk: 5%) and backward elimination (alpha risk: 0.1%) process.

#### Model verification and qualification

The robustness of the final PopPK model was qualified by goodness-of-fit (GOF) plots, quality criteria [bias, precision, or Absolute Average Fold Error (AAFE)], bootstrap analysis and visual predictive checks (VPCs) to ascertain the predictive power, model stability, and uncertainty in the parameter estimates. For the graphical assessment of the predictive ability of the model, 1000 simulated replicates of the observed dataset were generated, and the results were presented as time after the end of the doses (TAD) using prediction corrected VPC (pcVPC) and as baseline bodyweight using VPC method.

For the bootstrap analysis, 1000 replicate datasets were generated from the original dataset and the model was fit to each of these replicated datasets. Medians and corresponding non-parametric 95% confidence interval (CI) (2.5th to 97.5th percentiles) were assessed for each parameter based on the successfully minimized runs and compared to the parameter estimates of the final model.

#### Evaluation of individual exposures

After qualification, the model was used to calculate the individual exposures [maximal concentration (C_max)_ and area under the curve in the 2-week dosing interval (AUC_2W_)] for each patient treated with AVAL (20 mg/kg or 40 mg/kg q2w) after a single dose over 2 weeks i.e., 336 h (virtual sampling every 0.1 h). The infusion rate was administered stepwise: 1 mg/kg/h for 30 min, 3 mg/kg/h for 30 min, and 5 mg/kg/h for 30 min and then 7 mg/kg/h to deliver the planned amount of the drug. The total infusion duration for 20 mg/kg and 40 mg/kg was 3.71 and 6.57 h, respectively. Bodyweight at the time the patients received their last dose (20 or 40 mg/kg) was considered for IOPD patients whereas baseline bodyweight was considered for LOPD patients. Descriptive statistics on exposure parameters were reported by dose, and variables such as age, bodyweight, gender, race, disease type, and previous treatment with alglucosidase alfa.

#### Virtual patient simulation analysis for alternative dosing regimen

Exposure parameters of AVAL (C_max_ and AUC_2W_) were simulated with the final PopPK model to propose bodyweight dosing adjustments in pediatric patients. As limited number of patients were available for < 18 years, exposures were simulated by gender for selected staggered age (years) within each virtual population category. The equations, parameters, and variance-covariance matrix of the PopPK model was calculated through their corresponding bodyweight (kg) with the lowest and highest bodyweight set to the 5th and 95th percentiles as per Centre for Disease Control and Prevention (CDC) growth charts [22]. The virtual populations were generated using truncnorm package [23]. The simulations were performed using the mrgsolve package [24].

Simulations with different bodyweight cut-off (25, 30, 35 and 40 kg) were performed so as to identify the appropriate bodyweight cut-off achieving similar exposure of AVAL in adult patients across all groups. A total of 4018 virtual pediatric patients (1 to < 2 years, n = 1002; 2 to < 6 years, n = 1000; 6 to < 12 years, n = 1008; 12 to < 18 years, n = 1008) were generated for each cut-off simulation. Corresponding simulated exposure distributions were generated for ≥ 18 years (n = 1000, virtual patients) from the available adult patient data. Individual exposure parameters were computed after a single dose of 20 mg/kg for all virtual adult patients. Virtual pediatric patients were simulated at a single dose of 40 mg/kg (bodyweight <cut-off) and a single dose of 20 mg/kg (bodyweight ≥cut-off).

## Results

### Patient demographics and data inclusion

A total of 91 patients (LOPD, n = 75; IOPD, n = 16) were included and 2498 AVAL plasma concentration measurements were obtained for the analysis. After excluding measurements below the LLOQ (n = 241) and previously [18] identified outliers (n = 15), 2242 (LOPD, n = 2042; IOPD, n = 200) concentration-time points were included in the present analysis. Overall, the percentage of male participants (53.8%) was slightly higher than females, with a mean (SD) age and bodyweight of 39.2 (20.3) years, and 67.7 (26.3) kg, respectively. The demographic and baseline characteristics of the patients included in the final analysis are summarized in Table 2.

**Table 2:** Demographic and baseline characteristics of the patients included in the final analysis

Characteristic	LOPD, N = 75	IOPD, N = 16	Total, N = 91
**Gender, n (%)**			
Males	39 (52.0%)	10 (62.5%)	49 (53.8%)
Females	36 (48.0%)	6 (37.5%)	42 (46.2%)
**Ethnicity, n (%)**			
Caucasian	68 (90.7%)	8 (50%)	76 (83.5%)
Black	2 (2.7%)	0 (0.0%)	2 (2.2%)
Asian	3 (4.0%)	8 (50.0%)	11 (12.1%)
Other	2 (2.7%)	0 (0.0%)	2 (2.2%)
**Age [years, n (%)]**			
< 6	0 (0.0%)	4 (25.0%)	4 (4.4%)
≥ 6 to < 12	0 (0.0%)	12 (75.0%)	12 (13.2%)
≥ 12 to < 18	1 (2.0)	0 (0.0%)	1 (1.1%)
≥ 18 to 65	65 (86.7%)	0 (0.0%)	65 (71.4%)
≥ 65	9 (12.0%)	0 (0.0%)	9 (9.9%)
**Mean Age [Years, Mean (± SD) [Min- Max]]**	46 (15.1) [16.5–78.4]	6.9 (3.2) [1–11]	39.2 (20.3) [1-78.4]
**Body weight [Kg, Mean (± SD) [Min- Max]]**	75.9 (20.1) [38–129]	29.2 (15.4) [9.9–63.5]	67.7 (26.3) [9.9–129]
**Pre-treatment with alglucosidase alfa n (%)**			
Yes	61 (81.3%)	0 (0.0%)	61 (67.0%)
No	14 (18.7%)	16 (100.0%)	30 (33.0%)
**Initial dose, n (%)**			
5 mg/kg	8 (10.7%)	0 (0.0%)	8 (8.8%)
10 mg/kg	7 (9.3%)	0 (0.0%)	7 (7.7%)
20 mg/kg	60 (80.0%)	6 (37.5%)	66 (72.5%)
40 mg/kg	NA	10 (62.5%)	10 (11.0%)
**Alanine amino transferase [(IU/L), Mean (± SD) [Min- Max]]**	71.6 (51.7)^a^ [19–319]	140 (55.7) [47–231]	83.8 (58.6)^b^ [19–319]
**Aspartate amino transferase [(IU/L), Mean (± SD) [Min- Max]]**	72.1 (52.1)^a^ [23–285]	222 (125.0) [64–507]	98.8 (90.6)^b^ [23–507]
**Creatine kinase [(µmol/L), Mean (± SD [Min- Max])]**	164 (55.9)^a^ [83-3128]	1243 (643.0) [318–2607]	769 (583.0)^b^ [83-3128]

^a^N=74; ^b^N=90

IOPD, infantile-onset Pompe disease; LOPD, late-onset Pompe disease; NA, not applicable; SD, standard deviation

### Population pharmacokinetic model

#### Model development

For allometric bodyweight-based scaling, the PSM obtained with the previous LOPD dataset [17] was used as starting point and data of IOPD patients was added. It involved nine structural parameters: CL (linear clearance); V_max_, K_m_ (Michaelis-Menten parameters describing non-linear clearance); distribution volumes V1 (central compartment), V2, V3 (peripheral compartments); Q2, Q3 and Q_pc_ describing 2-way inter-compartmental clearance between V1 and V2, 1-way inter-compartmental clearance between V2 and V3 and low inter-compartmental clearance from V3 to V1 respectively. Similar to the previous LOPD PopPK analysis [18], the parameters Q2, V2, Q3, and V3 were fixed to avoid model overparameterization and non-identifiability.

Based on parsimony principle, the selected allometrized model included the time-varying bodyweight on CL, V_1_ and V_m_ parameters, thereby improving objective function value (OFV = 5295) as compared to model without allometric scaling (OFV = 5509). Despite extensive search (see summary in Table 1 in supplementary material) the best model didn’t include allometric factors on peripheral compartment parameters. The identified time-varying bodyweight showed an increase in CL (+ 41%), V_1_ (+ 28%), and V_m_ (+ 19%) in a patient weighing 100 kg and a decrease in CL (–56%), V_1_ (-45%), and V_m_ (-35%), in a patient weighing 27.3 kg versus a typical patient weighing 68.1 kg (CL, V_1_ and V_m_: 0.783 L/h, 3.29 L, and 11.8 mg/h respectively). The allometric scaling process allowed a decrease in the between-patient variability for all parameters (CL: up to 2 times lower) except Q_PC_.

The relationship between the individual estimates and the covariates was further investigated on the allometrized model using a forward/backward selection method. None of the investigated covariate, including disease type (IOPD versus LOPD), fulfilled the request criteria for selection except normalized CL_CRN_ on clearance (BCL_CRN_; ΔOFV = 15). When considering the minimum (50.2 mL/min/1.73 m^2^) and maximum (528 mL/min/1.73 m^2^) of BCL_CRN_ in the dataset, CL values were 0.781 L/h and 0.843 L/h, respectively. As compared to a typical patient (with a CL value of 0.812 and a BCL_CRN_ median value of 161 mL/min/1.73 m^2^), these values correspond to a limited effect of ± 4%. Consequently, BCL_CRN_ covariate was not retained in the present analysis due to insubstantial impact on CL parameters and in addition it was difficult to consistently compare the renal function through CL_CRN_ between adults and pediatric patients < 12 years. Therefore, following the allometric scaling process, no supplementary covariates were selected and allometrized PSM was considered as the final PopPK model. Inter-patient variability in model parameters, ranged between 13.6% and 52.4% for V1, CL, V_m_, K_m_ and up to 156% for Q_pc_. The residual (intra-individual) variability, modeled through a proportional error model was acceptable with a ~ 35% CV. The percentage of relative standard error of parameters (% RSE) were < 30% with none of the corresponding 95% CIs including zero (Table 3). The conditional number was 4.56 (< 1000) suggesting the model was not over-parameterized. The final PopPK parameters are presented in Table 3.

**Table 3:** Fixed and random effects parameter estimates for the population pharmacokinetic model

Population parameter	Estimate (CV %)	RSE (%)	[95%CI]	Bootstrap Median	Bootstrap [95% CI]
C_L_ (L/h)	0.808	3.33%	[0.755;0.862]	0.796	[0.674;0.874]
V_1_ (L)	3.37	2.21%	[3.22;3.52]	3.36	[3;3.57]
V_max_ (mg/h)	12	4.59%	[10.9;13.1]	12.2	[9.28;15.1]
K_m_ (µg/mL)	0.541	4.45%	[0.493;0.589]	0.55	[0.395;0.728]
Q_2_ (L/h)	0.254 (Fixed)	NA	NA	0.254 (Fixed)	NA
V_2_ (L)	296 (Fixed)	NA	NA	296 (Fixed)	NA
Q_3_ (L/h)	1.87 (Fixed)	NA	NA	1.87 (Fixed)	NA
V_3_ (L)	1.31 (Fixed)	NA	NA	1.31 (Fixed)	NA
Q_pc_ (L/h)	0.0157	11.60%	[0.0121;0.0194]	0.0134	[0.00826;0.0227]
Effect of WT on C_L_^a^	0.896	7.91%	[0.754;1.04]	0.889	[0.618;1.1]
Effect of WT on V_1_^b^	0.661	6.29%	[0.578;0.744]	0.652	[0.484;0.78]
Effect of WT on V_m_^c^	0.463	12%	[0.352;0.574]	0.472	[0.166;0.652]
**Inter-individual variability**					
ω² C_L_	0.0907 (30.8%)	18.50%	[0.0578;0.124] (7.56%)	0.0896	[0.0573;0.136]
ω² V_1_	0.0184 (13.6%)	35.20%	[0.00569;0.031] (28.3%)	0.0189	[0.00476;0.0388]
ω² V_m_	0.118 (35.4%)	25.40%	[0.0593;0.177] (30.4%)	0.117	[0.0201;0.236]
ω² K_m_	0.243 (52.4%)	27.90%	[0.11;0.376] (29.6%)	0.214	[0.0702;0.417]
ω² Q_pc_	1.23 (156%)	26.20%	[0.599;1.86] (30.6%)	1.72	[0.492;3.48]
**Residual variability**					
σ²	0.12 (34.6%)	2.43%	[0.114;0.125]	0.116	[0.0967;0.136]

%RSE, relative standard error (100% * SE / Estimate); θ and ω are the PopPK parameters; ω², variance of their associated inter-individual variability; NA, not applicable

^a^the expression of the linear clearance (CL) including covariates effects is: CL = TVCL x (WT/70.5)** θ10. WT is the weight and 70.5 the median of weight values corresponding to 70.5 kg; ^b^the expression of the linear clearance (CL) including covariates effects is: V1 = TVV1 x (WT/70.5)** θ11. WT is the weight and 70.5 the median of weight values corresponding to 70.5 kg; ^c^the expression of the non-linear clearance Vm parameter including covariate effect is: Vm = TVVm x (WT/70.5)** θ12. WT is the weight and 70.5 the median of weight values corresponding to 70.5 kg

#### Model verification and qualification

The final model adequately described the data. There was no important systematic deviation or major bias as demonstrated with the goodness-of-fit (GOF) plots (Fig. 1), where population (PRED) and individual predictions (IPRED) showed lack of any bias when plotted against observed (OBS) data, and with no obvious trends in plots of conditional weighted residuals (CWRES) versus population predictions and time after dose (Fig. 1 and additional GOF plots in supplementary material).

**Fig. 1a:**
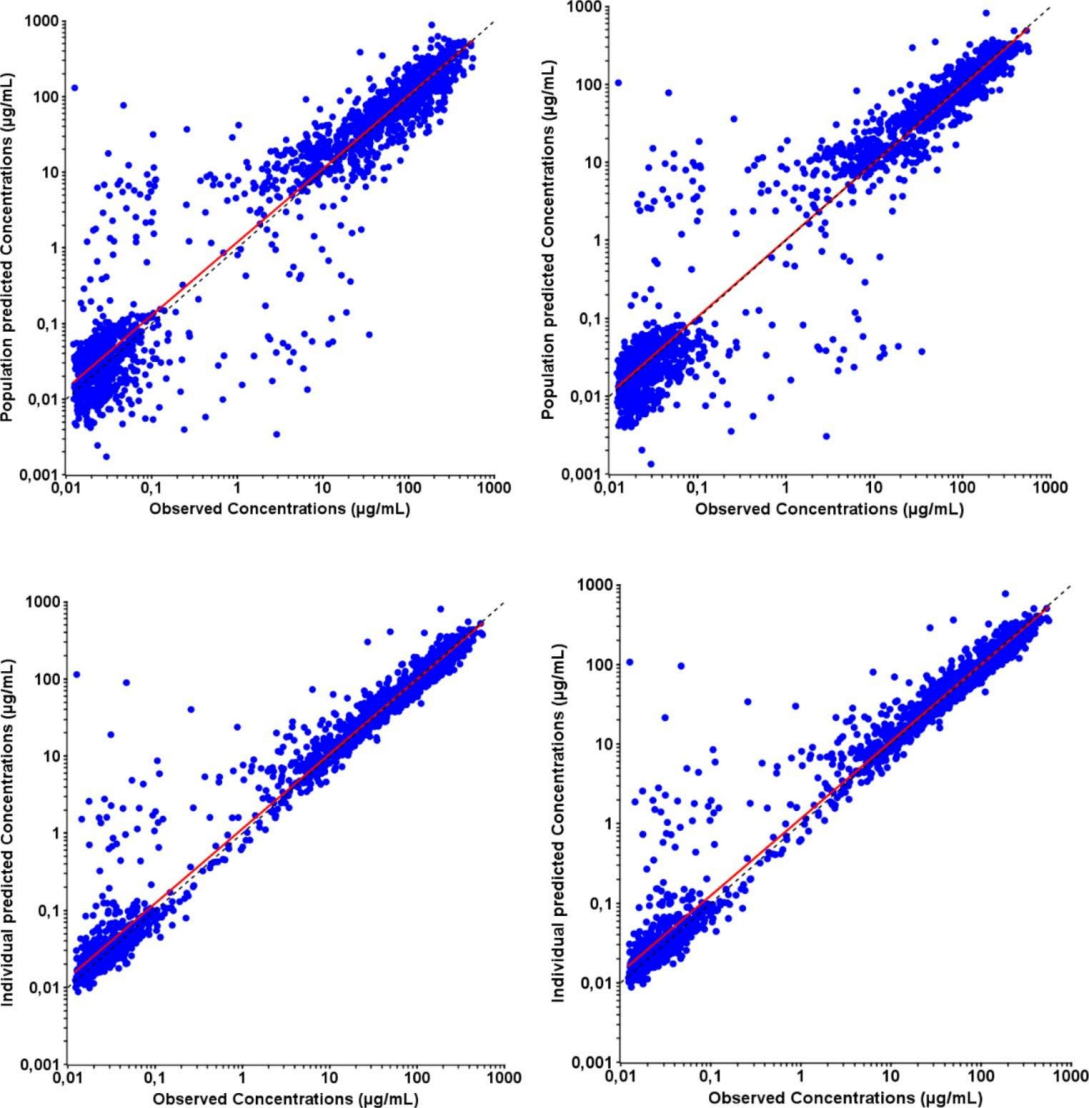
PRED and IPRED versus observed concentrations goodness-of-fit plots (Logarithmic scale)

**Fig. 1b:**
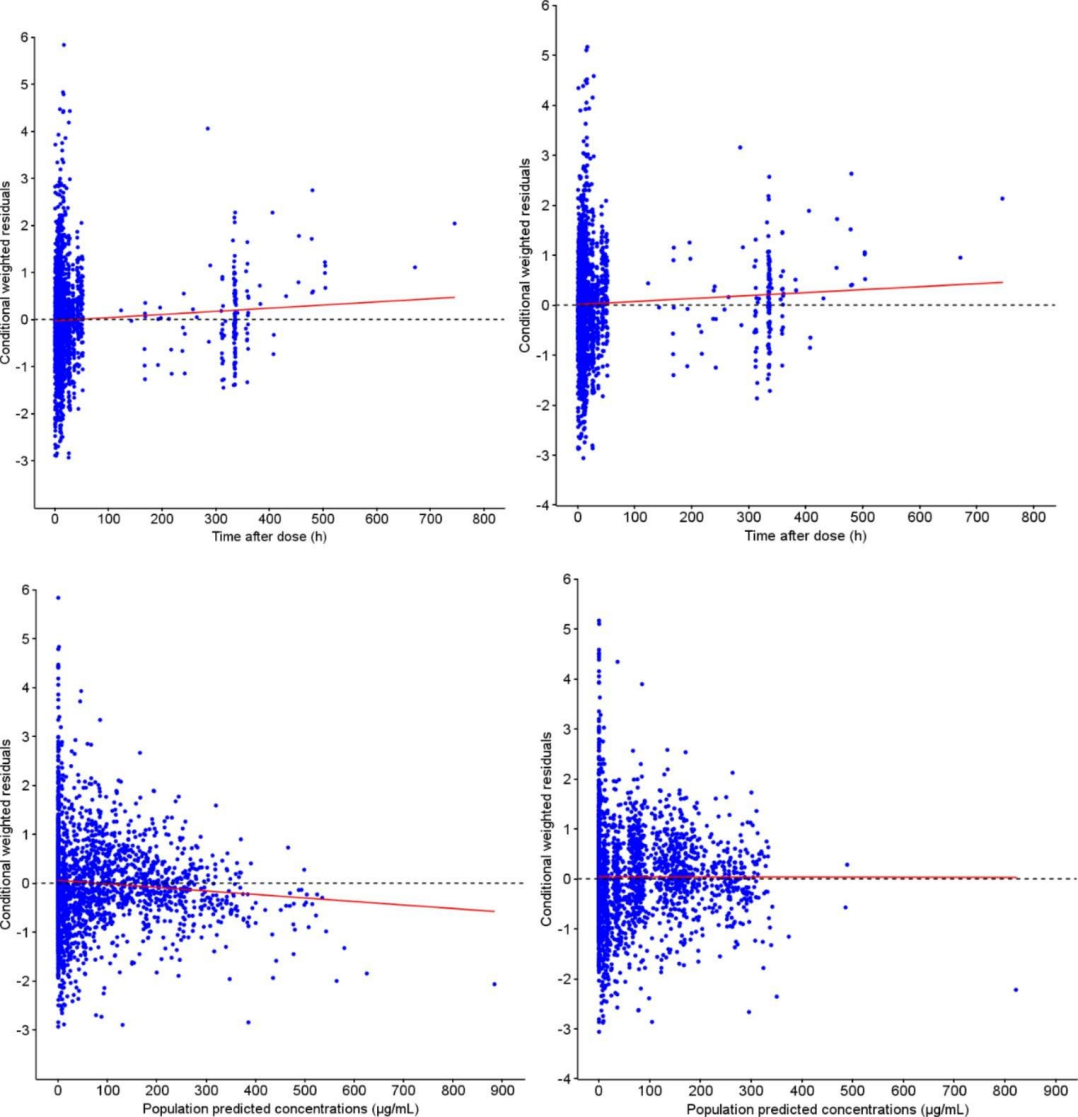
CWRES versus time after dose and versus PRED plots. Left: before covariate inclusion. Right: after covariate inclusion. Dashed black line: zero line – Red line: regression line. GOF, goodness of fit; CWRES, conditional weighted residues; IPRED, individual prediction; PRED, population prediction

The bias was low but significantly different from zero for PRED versus OBS and IPRED versus OBS. The AAFE values and precision estimates for OBS versus PRED and OBS versus IPRED were 1.73, 1.36 and 53.6%, 42.6% respectively (see Table 2 in supplementary material for more details). Furthermore, the correlation characteristics (r ≥ 0.91) were good in both cases. The allometric bodyweight-based scaling strongly increased the model quality as the quality criteria were closer between LOPD and IOPD patients in the model than in the PSM before allometric scaling.

In the bootstrap analysis, out of 1000 replicates, 846 runs minimized successfully. The 95% CI were reasonably narrow and median bootstrap values for parameters were in good agreement (negligible deviation) with the PopPK parameters, highlighting the robustness of the model (Table 3).

As presented in Fig. 2a and b, the VPC assessment suggested a reasonable predictive capacity of the model as the majority of simulated AVAL concentrations were in the 90% CI, largely overlapping with the observed values for both the high and low range drug concentrations.

**Fig. 2a:**
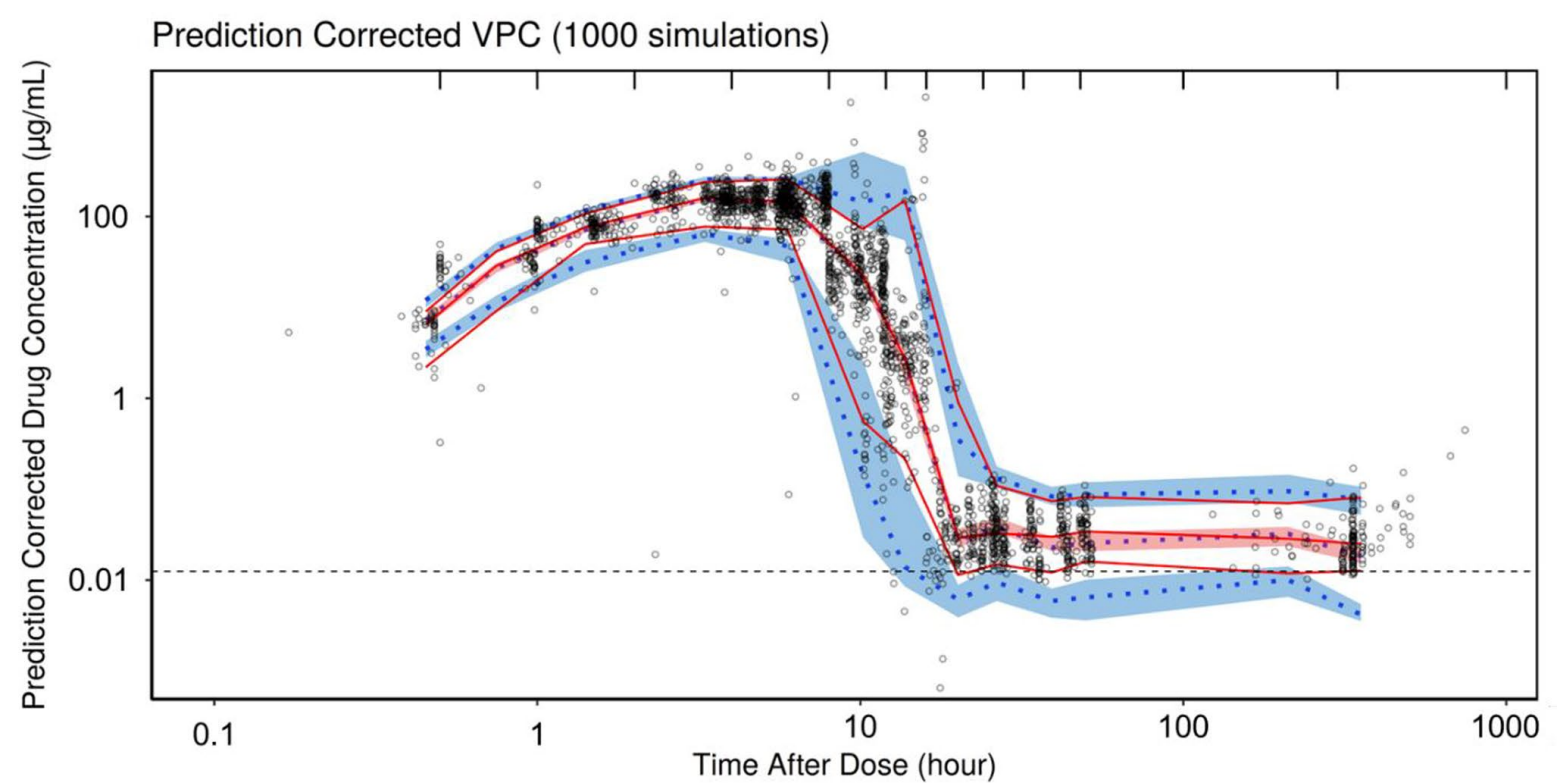
Prediction corrected visual predictive check logarithmic plots for TAD

**Fig. 2b:**
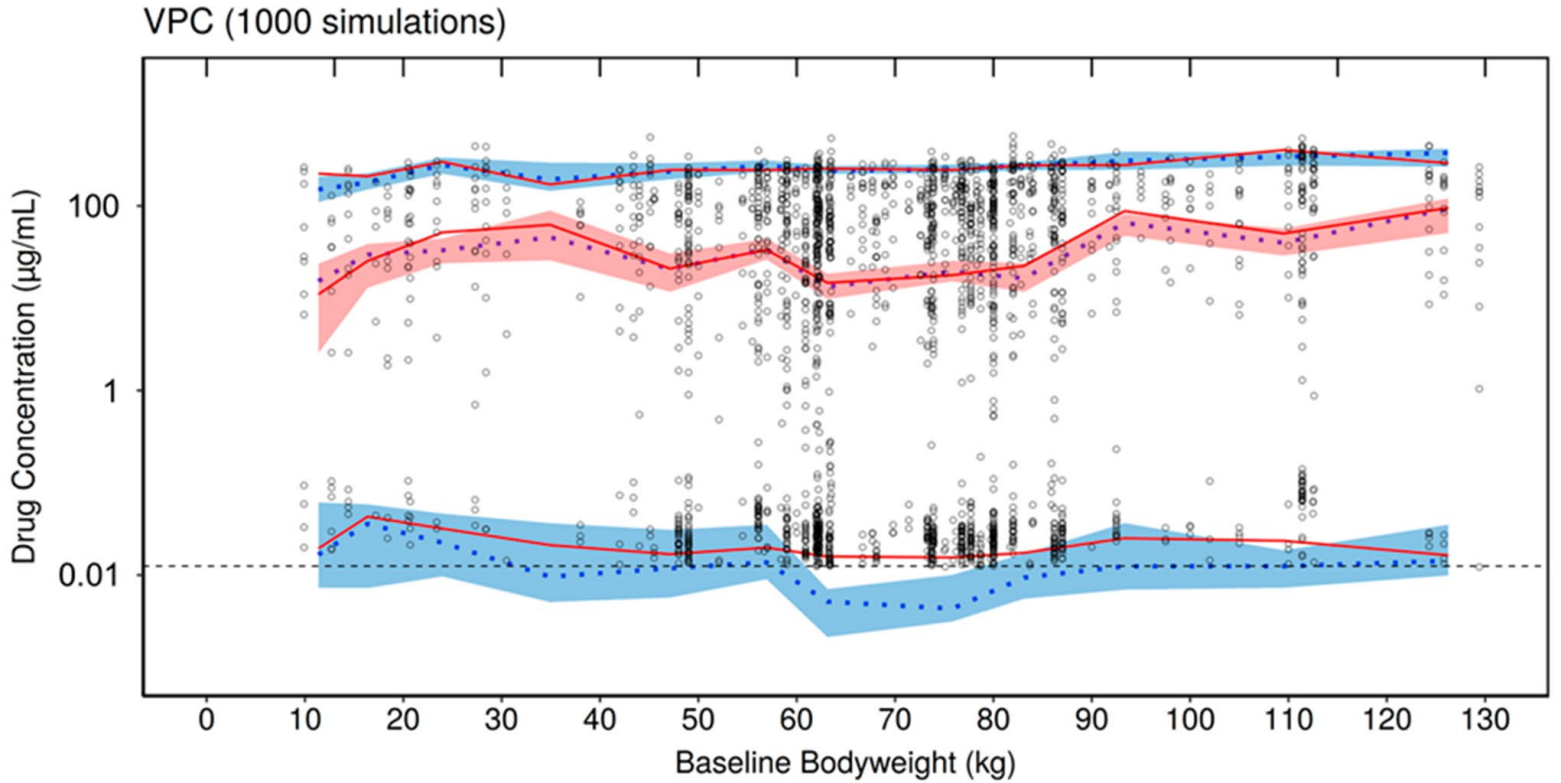
Visual predictive check semi-logarithmic plots by bodyweight. The black circles correspond to the observed data; the red solid lines correspond to the median, 5th and 95th percentile of observed data; the blue dotted lines correspond to the median, 5th and 95th percentile of simulated data; the shaded areas correspond to the CI95 of the percentiles of the simulated data; the dashed blue line corresponds to the LOQ (0.0125 µg/mL)

#### Exposure parameters

Based on the individual simulations, the variability (CV%) of AVAL for C_max_ and AUC_2W_ was 21.7% and 23.0% (40 mg/kg; n = 10) and 18.3% and 26.5% (20 mg/kg; n = 76) respectively. Both C_max_ and AUC_2W_ slightly increased with increase in bodyweight (Table 4). The median AUC_2W_ and C_max_ (20 mg/kg) was − 28.4% and − 20.5% respectively lower for < 50 kg group and, + 14.5% and + 16.7% higher for ≥ 100 kg group (reference group: 50–100 kg). Since age and bodyweight covariates were correlated, a similar trend was observed for stratification by age, and type of disease (IOPD or LOPD). None of the other covariate stratifications was identified as showing a meaningful impact on AVAL exposures. Descriptive statistics of the AVAL exposures are presented in Table 4.

**Table 4:** Exposure parameters of avalglucosidase alfa (20 mg/kg and 40 mg/kg dose)

Variable	20 mg/kg				40 mg/kg		
	n		C_max_ (µg/mL)	AUC_2W_ (µg/mL)	n	C_max_ (µg/mL)	AUC_2W_ (µg/mL)
**All patients**	76^a^		[261;139–378]	[1140; 463–2149]	10^b^	[275; 195–382]	[1872; 1247–2620]
**Disease type**							
IOPD	6		[157; 139–256]	[591; 463–1089]	10	[275; 195–382]	[1872; 1247–2620]
LOPD	70		[266; 160–378]	[1164; 737–2149]	0	NA	NA
**Age** ^**c**^ **(years)**							
< 6	1	139		463	3	[216; 195–250]	[1419; 1247–1584]
6–11	5	[157; 151–256]		[591; 576–1089]	7	[320; 232–382]	[2181; 1536–2620]
12–17	1	258		1013	0	NA	NA
18–64	59	[268; 189–378]		[1141; 737–2149]	0	NA	NA
≥ 65	10	[265; 160–368]		[1264; 824–2086]	0	NA	NA
**Body weight** ^**d**^ **(kg)**							
< 50	11	[209; 139–256]		[833; 463–1089]	9	[250; 195–381]	[1584; 1247–2620]
50–100	55	[263; 160–368]		[1164; 749–2086]	1	382	2449
≥ 100	10	[307; 273–378]		[1333; 997–2149]	0	NA	NA
**Gender**							
Female	34	[254; 139–366]		[1053; 463–2149]	5	[232; 195–328]	[1536; 1247–2277]
Male	42	[271; 151–378]		[1188; 576–2086]	5	[275; 216–382]	[1872; 1419–2620]
**Ethnicity**							
Asian	6	[231; 151–279]		[933; 591–1175]	5	[275; 232–381]	[1872; 1536–2620]
Black	2	[255; 255–278]		[1204; 1204–1264]	0	NA	NA
Caucasian	68	[265; 139–378]		[1141; 463–2149]	5	[216; 195–382]	[1419; 1247–2449]
**Alglucosidase alfa treatment**							
Naïve	58	[266; 189–368]		[1179; 737–2149]	0	NA	NA
Pre-treated	18	[238; 139–378]		[1072; 463–1764]	10	[275; 195–382]	[1872; 1247–2620]
**CL**_**CRN**_ **(mL/min/1.73 m²)**							
30:60	1	[265; 265–265]		1402	0	NA	NA
60:90	4	[301; 160–368]		[1459; 824–2086]	0	NA	NA
≥ 90	71	[259; 139–378]		[1140; 463–2149]	10	[275; 195–382]	[1872; 1247–2620]
**Albumin (g/L)**							
< 45^e^	32	[238; 139–378]		[1038; 463–2086]	3	[250; 232–275]	[1584; 1536–1872]
≥ 45^e^	44	[273; 189–366]		[1179; 737–2149]	7	[320; 195–382]	[2181; 1247–2620]
**ALP (IU/L)**							
< 73^e^	38	[255; 189–378]		[1118; 737–1764]	0	NA	NA
≥ 73^e^	38	[265; 139–368]		[1189; 463–2149]	10	[275; 195–382]	[1872; 1247–2620]
**ALT (IU/L)**							
< 66^e^	41	[268; 160–378]		[1160; 737–2149]	1	250	1584
≥ 66^e^	35	[256; 139–355]		[1089; 463–1846]	9	[275; 195–382]	[1872; 1247–2620]
**AST (IU/L)**							
< 64^e^	42	[268; 160–378]		[1164; 737–2149]	0	NA	NA
≥ 64^e^	34	[255; 139–355]		[1053; 463–1846]	10	[275; 195–382]	[1872; 1247–2620]
**Bilirubin (µmol/L)**							
< 6.8^e^	33	[252; 139–366]		[1053; 463–2149]	7	[250; 195–382]	[1584; 1247–2620]
≥ 6.8^e^	43	[273; 160–378]		[1189; 737–2086]	3	[314; 275–320]	[2116; 1872–2181]
**Creatine Kinase (IU/L)**							
< 580^e^	40	[271; 139–378]		[1188; 463–2149]	1	250	1584
≥ 580^e^	36	[256; 151–355]		[1053; 591–1566]	9	[275; 195–382]	[1872; 1247–2620]

Descriptive statistics are [Median; minimum-maximum]

ALP, alkaline phosphatase; ALT, alanine transaminase; AST, aspartate aminotransferase; CL_CRN,_ creatinine clearance; IOPD, infant-onset Pompe disease; LOPD, late-onset Pompe disease; NA: not applicable

^a^includes 69 adult patients, 1 adolescent patient and 6 patients ≤ 11 years; ^b^includes 10 patients ≤ 11 years; ^c^age at the time patients received their first 20 or 40 mg/kg dose (different to the age at baseline for patients switching from 5 or 10 m/kg to 20 mg/kg in study NCT02032524); ^d^body weight used for exposure computation, i.e., body weight at the time patients received their first 20 mg/kg dose for LOPD patients and body weight at the time patients received their last dose for IOPD patients; ^e^median of baselines calculated from the total data

#### PK simulations by bodyweight

**Fig. 3:**
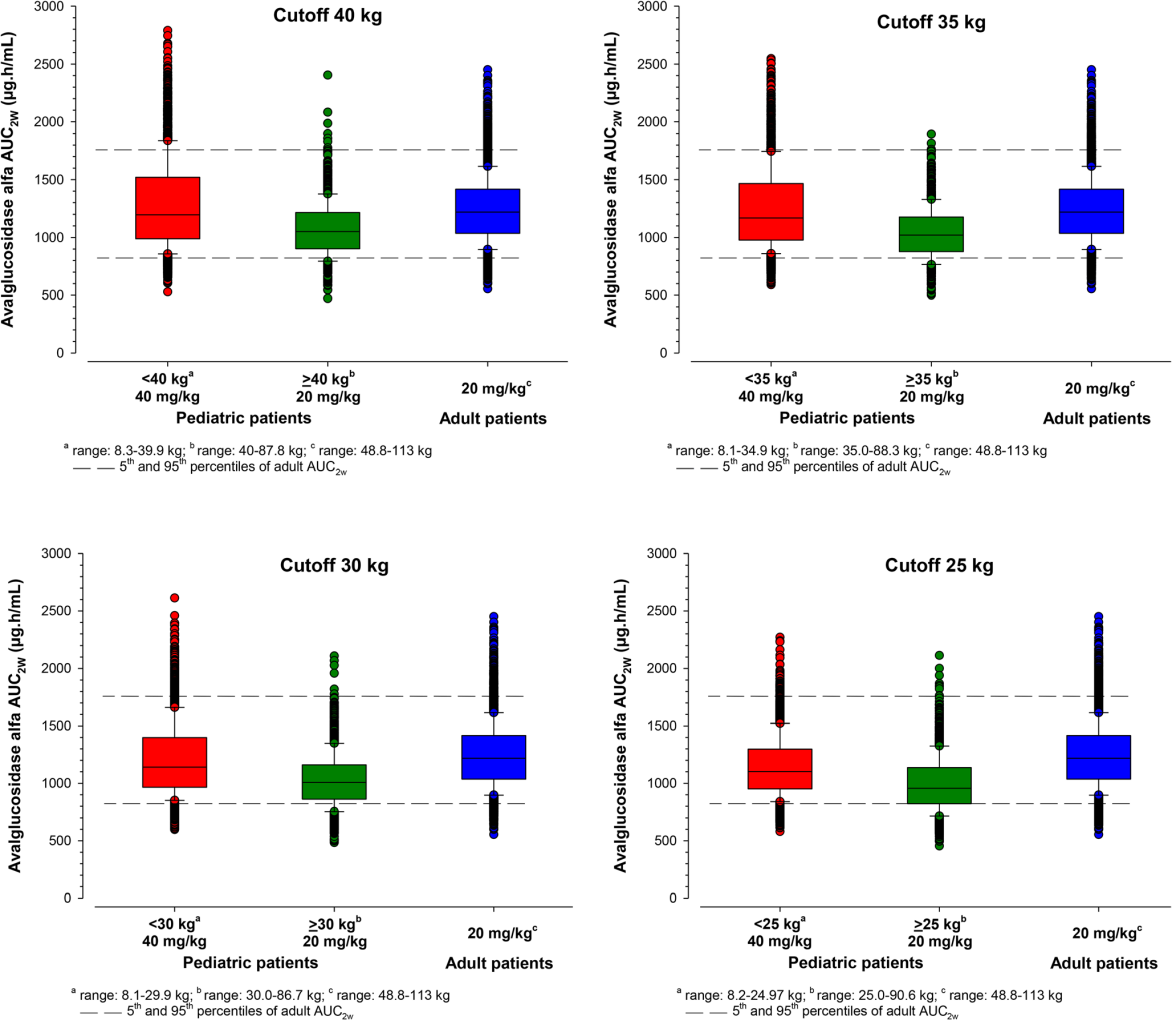
Simulated avalglucosidase alfa AUC2W by bodyweight cutoff (25, 30, 35 and 40 kg) and dose in pediatric versus adult virtual patients (10th, 25th, 50th, 75th, and 90th percentiles, and individual values)

**Fig. 4:**
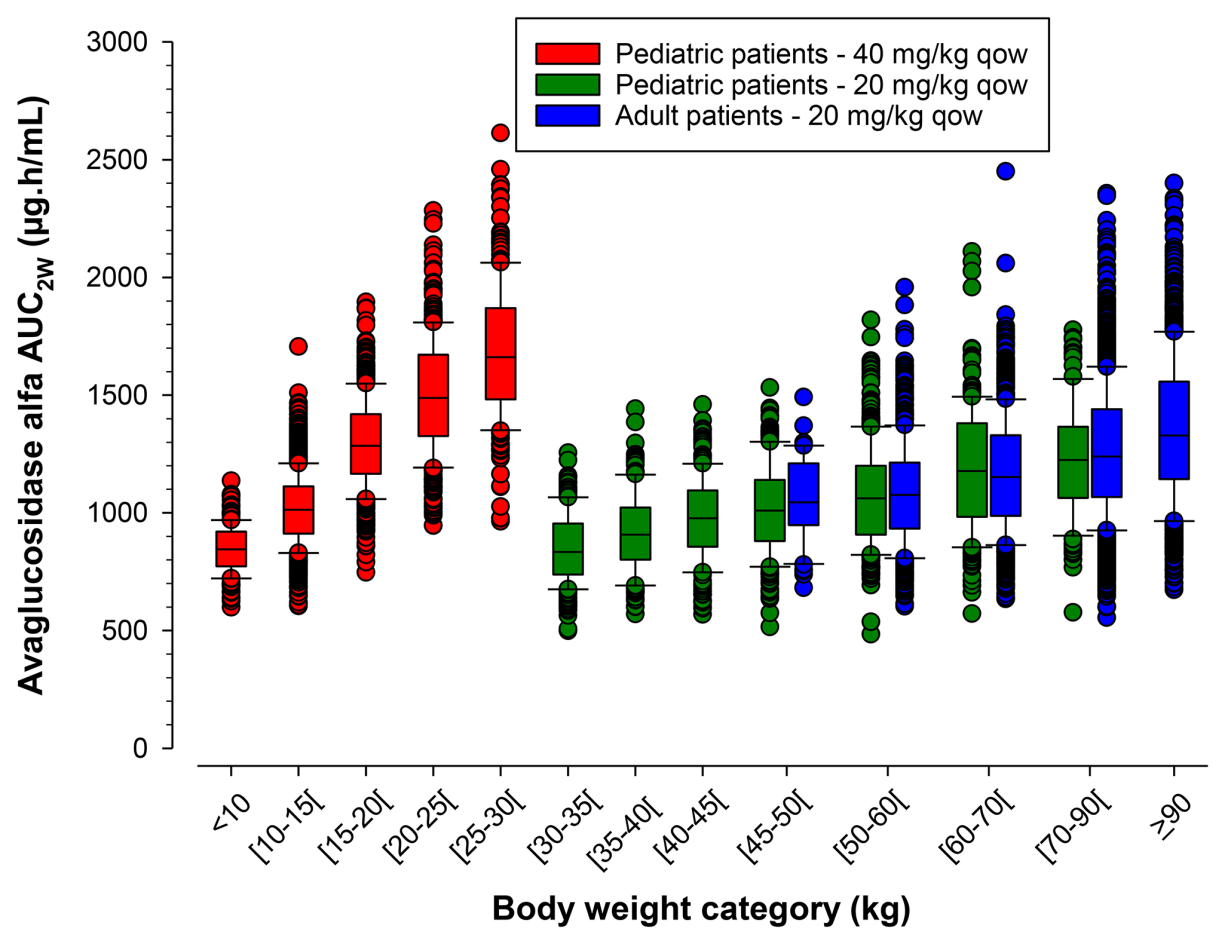
Simulated avalglucosidase alfa AUC_2W_ by body weight category following 40 mg/kg (< 30 kg) or 20 mg/kg (≥ 30 kg) in pediatric versus adult virtual patients (10th, 25th, 50th, 75th, and 90th percentiles, and individual values)

The distribution of bodyweight generated as per age categories in virtual populations was found to be in accordance with the CDC growth chart (see Figure in supplementary material). The median exposures following 20 mg/kg in different virtual pediatric age groups were lower, compared with adult population (C_max_: 12–55%; AUC_2W_: 13–63%). PK simulations by bodyweight indicated that 40 mg/kg for pediatric patients resulted in a similar median AUC_2W_ in pediatric patient with bodyweight < 40 kg, < 35 kg or 30 kg compared to adult patients receiving the 20 mg/kg dosing regimen (Fig. 3). The AUC_2W_ at 40 mg/kg were slightly higher for the 40 and 35 kg cut-off compared with adults for the extreme values. These results suggested that 40 mg/kg and 20 mg/kg dosing regimen in pediatric patients with bodyweight < 30 kg and ≥ 30 kg, respectively allowed to achieve similar AVAL exposure (based on AUC_2W_) to adult patients receiving a 20 mg/kg dosing regimen (Fig. 4).

## Discussion

The present study was conducted to address the FDA request to supplement the adolescent and adult LOPD data with pediatric patient data to provide supporting data for dosing adapted bodyweight in LOPD patients ≥ 1 year. The FDA supports ‘complete extrapolation of efficacy’ approach of adult PK data to the pediatric population if it can be assumed that children and adults have similar disease progression, response to treatment, and exposure-response [13]. Using this approach, the present analysis identified dose in pediatric population by matching systemic exposures between adult and pediatric population supported by safety data at the identified dose [17, 25].

PopPK modeling is one of the best methods to analyze limited and unbalanced datasets. This approach explores the influence of various covariates such as bodyweight and age to understand variable drug responses in children [26]. Hence, PK simulations comparing exposures across different age and bodyweight groups were conducted to propose alternative dosing regimens based on a bodyweight cut-off. Allometric scaling is a very commonly used approach for extrapolating PK data from adult to pediatric patients [27, 28]. Since previous analysis included only one pediatric patient with LOPD (age: 16 years), allometric modeling was unsuccessful as bodyweight effect could not be evidenced [18]. In the present study, PopPK model was built using data from both IOPD patients (1–12 years) and LOPD patients. Since the bodyweight of the pediatric population increased during the clinical study, a time-varying bodyweight was used for the allometric scaling process.

The selected model included the time-varying bodyweight on CL, V1 and V_m_ parameters. This model accurately predicted serum concentrations of AVAL which was demonstrated at each step of the model building process. The allometric scaling improved OFV versus non-allometrized model and reduced the between-patient variability for CL, V1 and Vm.

The bodyweight had two opposite effects on the computed exposure parameters. Firstly, increase in bodyweight led to higher total clearance (see supplementary material) and consequently lower C_max_ and AUC_2W_. On the other hand, weight-based dose regimen increases the amount of injected AVAL with increase in bodyweight followed by higher C_max_ and AUC_2W_. As a result, there was a small increase in C_max_ and AUC_2W_ as patient’s bodyweight increased. Rather than a pure bodyweight effect and due to the specificity of the dataset (adult plus pediatric patients), the exposure parameters differences among patients could be related to their age. This was further explored by analyzing the bodyweight normalized apparent clearance (calculated by the following equation) considering the computed individual AUC_2W_ for both 20 mg/kg and 40 mg/kg doses.

$$\frac{CL}{WT}=\frac{Dose}{AU{C}_{2W} \times Body weight}$$

The apparent CL/WT was decreased with increase in age of pediatric patient. Similar observations have been reported in the PK analysis of other ERTs for lysosomal storage diseases, suggesting a greater cellular uptake of AVAL for the youngest patients [29, 30]. This could be due to an up-regulation of the systemic M6P receptor in children as compared to adults, as reported in preclinical studies on rats [31]. However, anticipating a direct link between the possible greater cellular uptake and a better clinical response for pediatric versus adult patients receiving the same doses is hazardous. An increased organ/bodyweight ratio was observed in children compared to adults for liver, spleen, and kidney [32]. This may increase the proportion of the clearance of AVAL by non-targeted tissues/organs (sink effects) in pediatric patients. Moreover, the efficiency of cellular uptake and targeting to lysosomes for AVAL may decrease with disease severity [33]. All these considerations hinder any anticipation of the potential differences between adults and pediatric patients receiving the same dose (in mg/kg) for the targeted tissue(s)/organ(s).

Recent studies have shown that higher doses of alglucosidase alfa are safe and may lead to better clinical outcomes in patients with IOPD or pediatric LOPD, owing to almost complete enzyme deficiency in such patients [34]. In addition, a multicentre observational cohort study from the European Pompe Consortium revealed that there was a significant improvement in survival and walking ability in patients with classic IOPD treated with the high dose alglucosidase alfa (40 mg/kg/week) versus standard recommended dose (20 mg/kg q2w) [35]. Moreover, currently there are no clinical studies available with AVAL/alglucosidase that showed if there is a relationship between efficacy and bodyweight or exposure and efficacy. Mini-COMET is the only available study establishing the safety of AVAL at 40 mg/kg dose in paediatric IOPD patients [17].

Simulation results pointed out that a dosing regimen of 40 mg/kg for pediatric patients resulted in a similar median AUC_2w_ with bodyweight < 30 kg versus adult patients receiving the 20 mg/kg. The individual AUC_2w_ of patients < 30 kg and ≥ 30 kg receiving 40 mg/kg and 20 mg/kg respectively were within the range of adult AUC_2w_. Recently, using the same virtual population and model described in this manuscript, reviewers from USFDA published their own analysis which corroborate our dosing choice for patients with bodyweight < 30 kg [36]. The ongoing Baby-COMET (NCT04910776) trial is evaluating the effects of AVAL 40 mg/kg qow for 52 weeks on overall and ventilator-free survival in IOPD treatment-naïve patients of age up to 12 months at enrollment [37].

## Conclusions

To conclude, the PopPK model including IOPD pediatric patients developed for IV administered AVAL showed a good agreement between model-predicted and observed plasma concentrations. PK simulations conducted on the basis of this model provided supporting data for the currently approved US labelling for dosing adapted bodyweight in LOPD patients ≥1 year by USFDA. These results will be validated with data from new studies that are underway for IOPD patients.

### Electronic supplementary material

Below is the link to the electronic supplementary material.

Supplementary Material 1

## Abbreviations

AAFE: Absolute Average Fold Error
AVAL: Avalglucosidase alfa
CDC: Centres for Disease Control and Prevention
CI-MPR: Cation independent mannose 6-phosphate receptor
CL: Clearance
CWRES: Conditional weighted residual
EMA: European Medicines Agency
ERT: Enzyme replacement therapy
FOCE: First-order conditional estimation
GAA: Acid alpha glucosidase
GCP: Good clinical practice
GOF: Goodness-of-fit
GSD: Glycogen storage disorder
IOPD: Infantile-onset Pompe disease
IPRED: Individual prediction
LLOQ: Lower limit of quantification
LOPD: Late-onset Pompe disease
6MP: Mannose-6-phosphate
OFV: Objective function value
pcVPC: Prediction corrected visual predictive checks
PopPK: Population pharmacokinetic
PRED: Population prediction
PSM: PharmacoStatistical model
TAD: Time after the end of the doses
USFDA: United States Food and Drug Administration
VPC: Visual predictive check
WT: Weight
