# Supplementary material for: Population pharmacokinetic modeling and dosing simulation of avalglucosidase alfa for selecting alternative dosing regimen in pediatric patients with late-onset pompe disease
Source: J Pharmacokinet Pharmacodyn. 2023 Aug 3;50(6):461–74. doi: 10.1007/s10928-023-09874-8 (PMC10673948; doi:10.1007/s10928-023-09874-8)
Supplement: Supplementary file 1 — Supplementary Material 1 [file 10928_2023_9874_MOESM1_ESM.docx]

**Supplementary materials**

Comparison of CDC growth chart and body weight generated in virtual populations


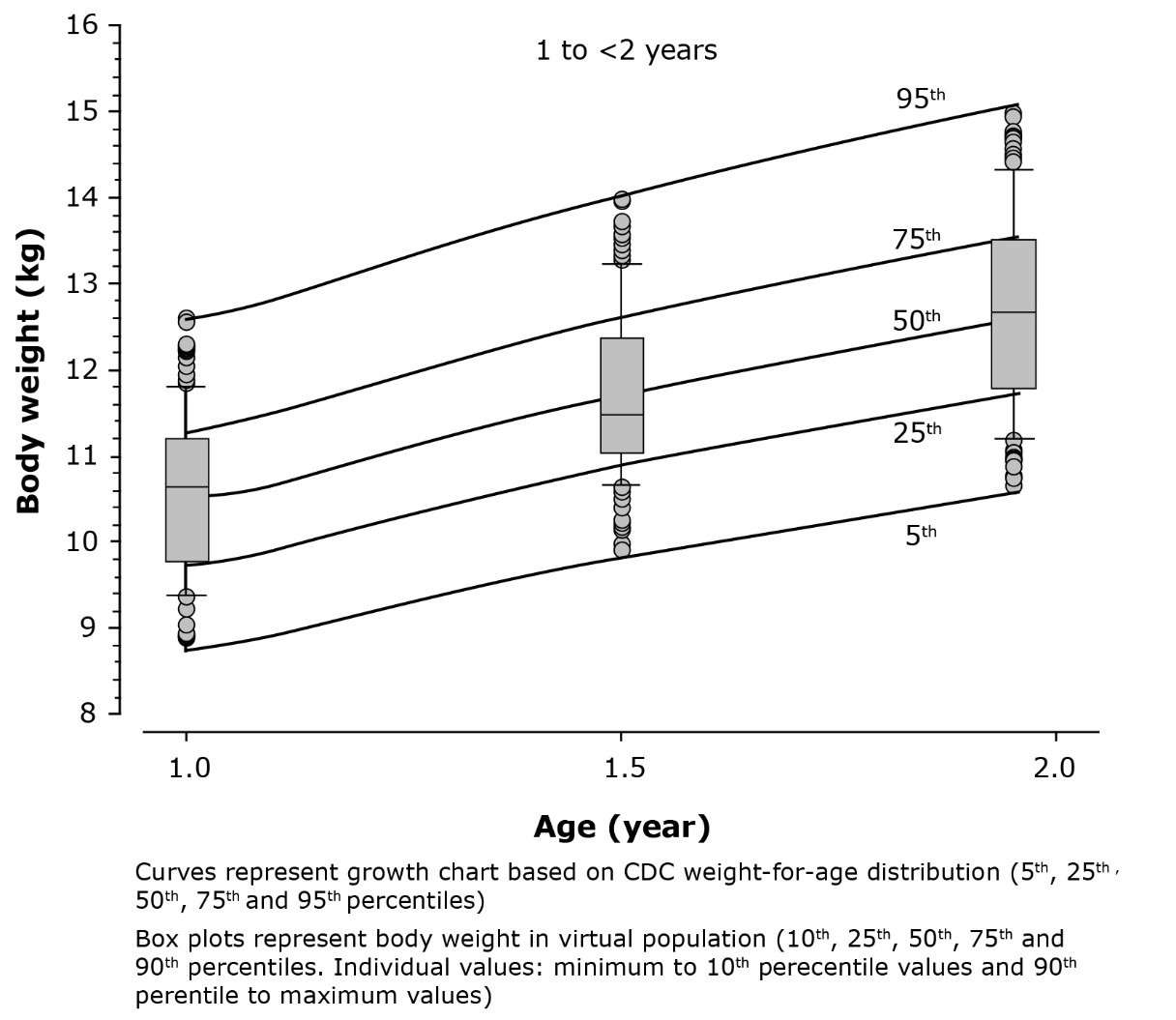


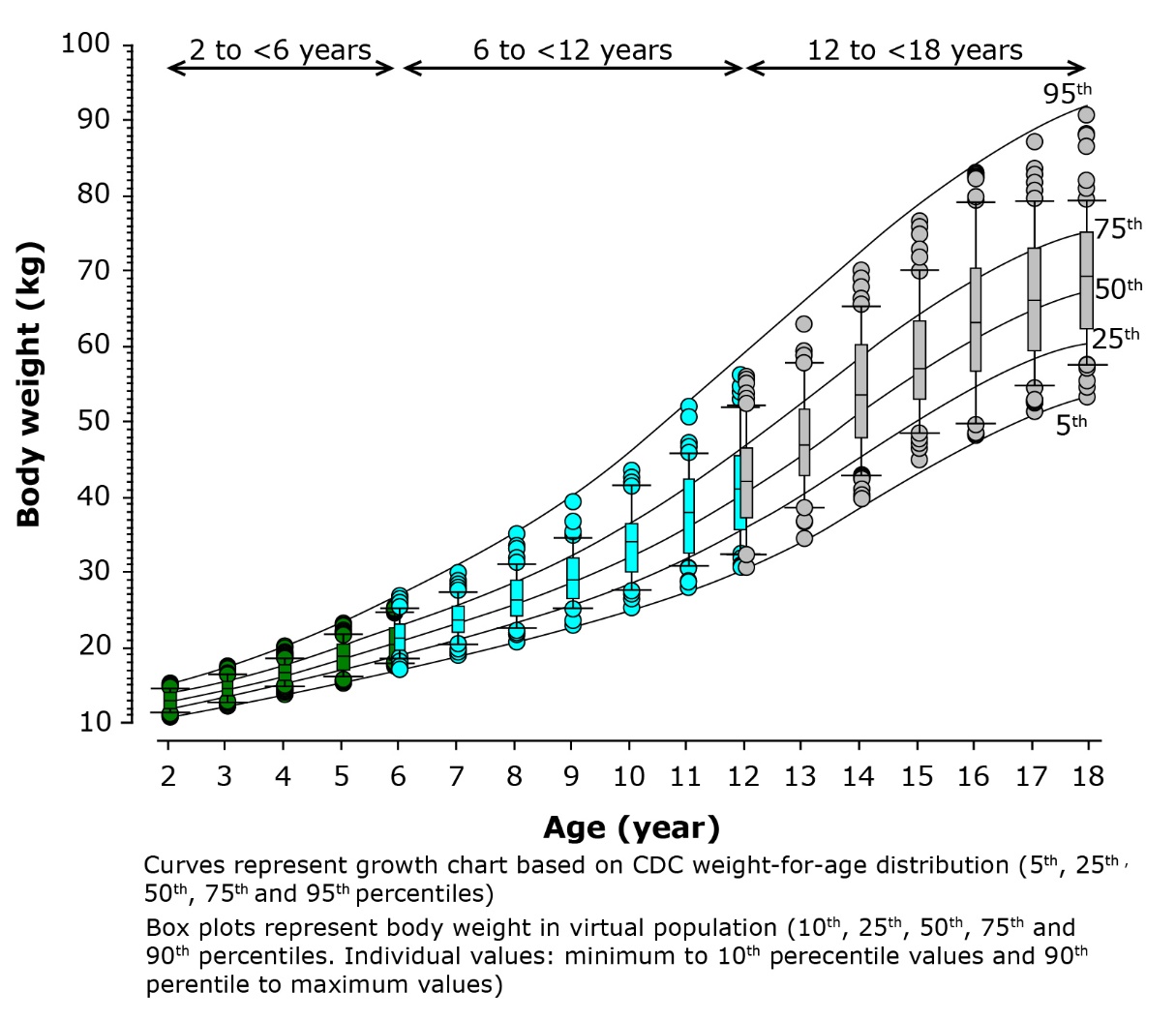


**Additional GOF plots**

PRED versus OBS before (left) and after (right) covariate inclusion – Logarithmic scale

| **Legend: Dashed black line: zero line – Red line: regression line** | |
| --- | --- |
| LOPD patients (n=75) | |
| 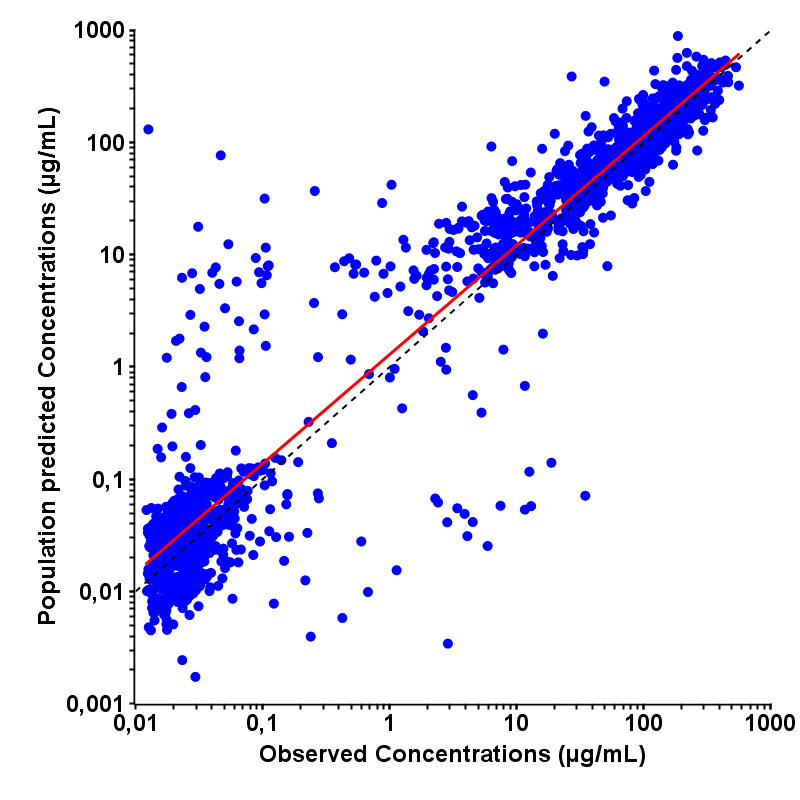 | 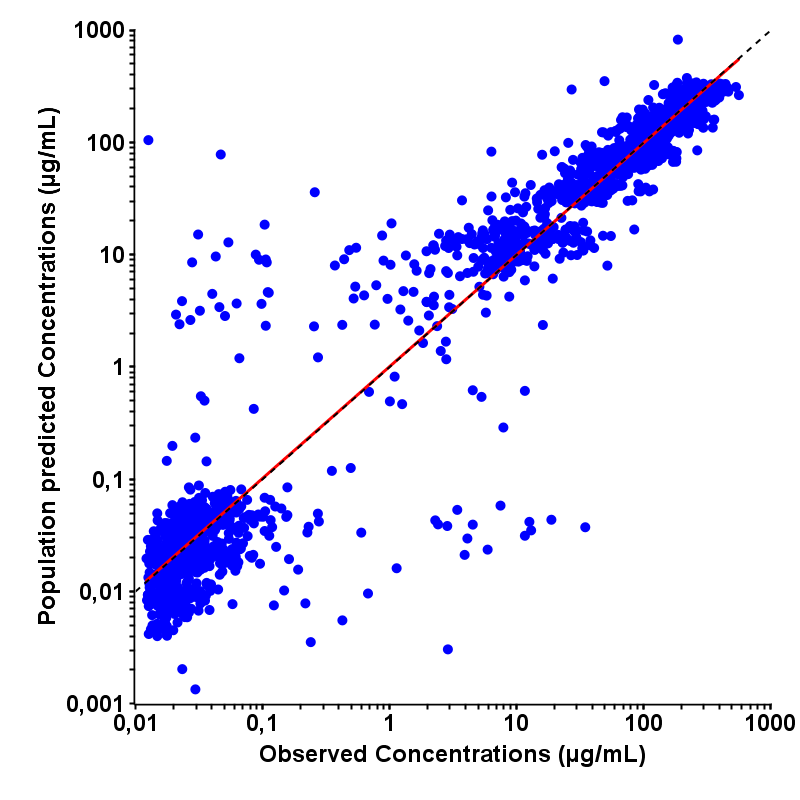 |
| IOPD patients (n=16) | |
| 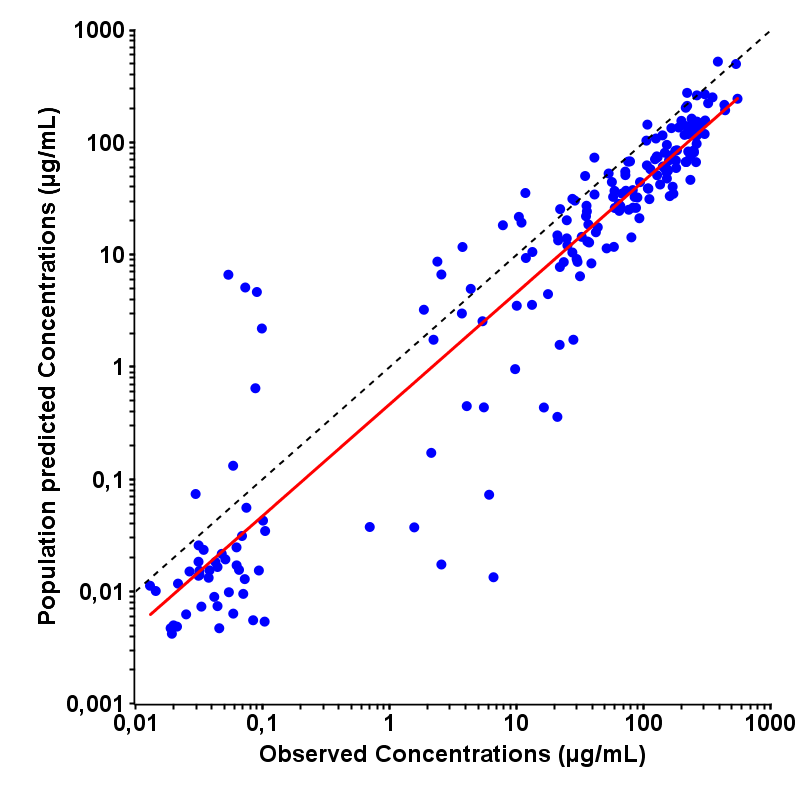 | 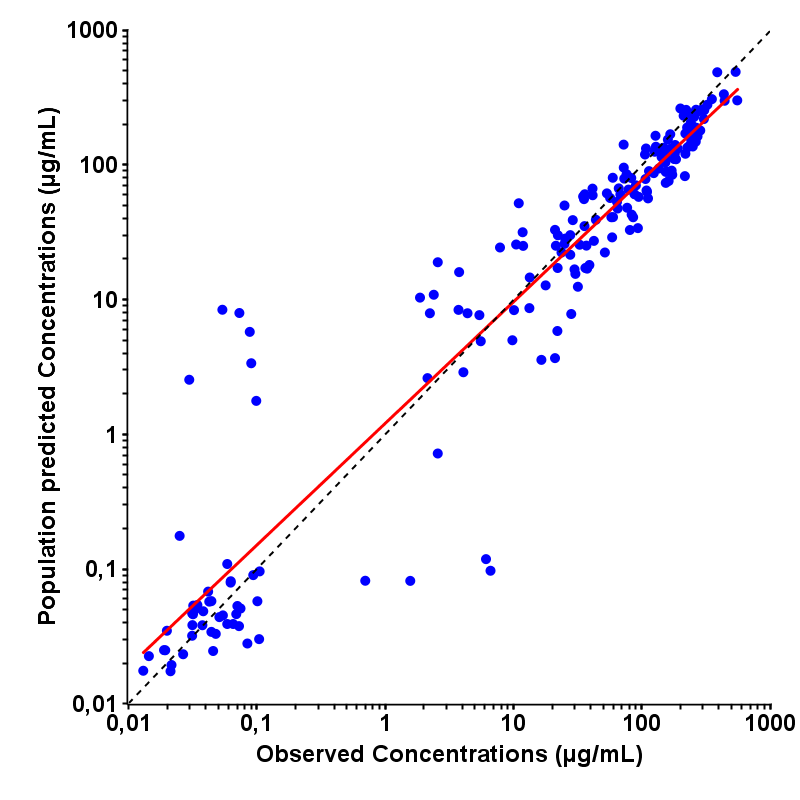 |

IPRED versus OBS before (left) and after (right) covariate inclusion – Logarithmic scale

| **Legend: Dashed black line: zero line – Red line: regression line** | |
| --- | --- |
| LOPD patients (n=75) | |
| 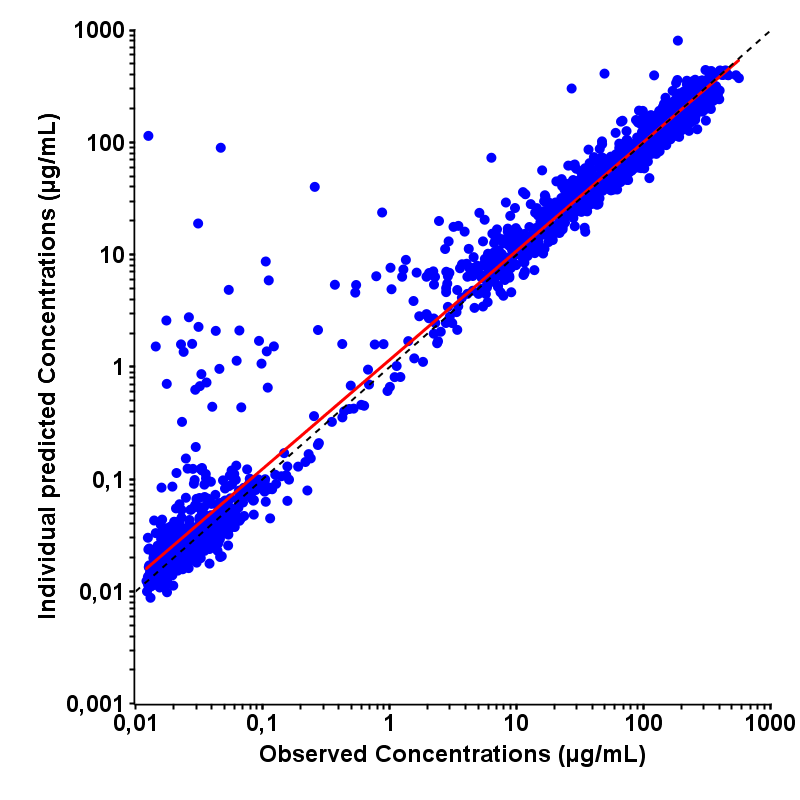 | 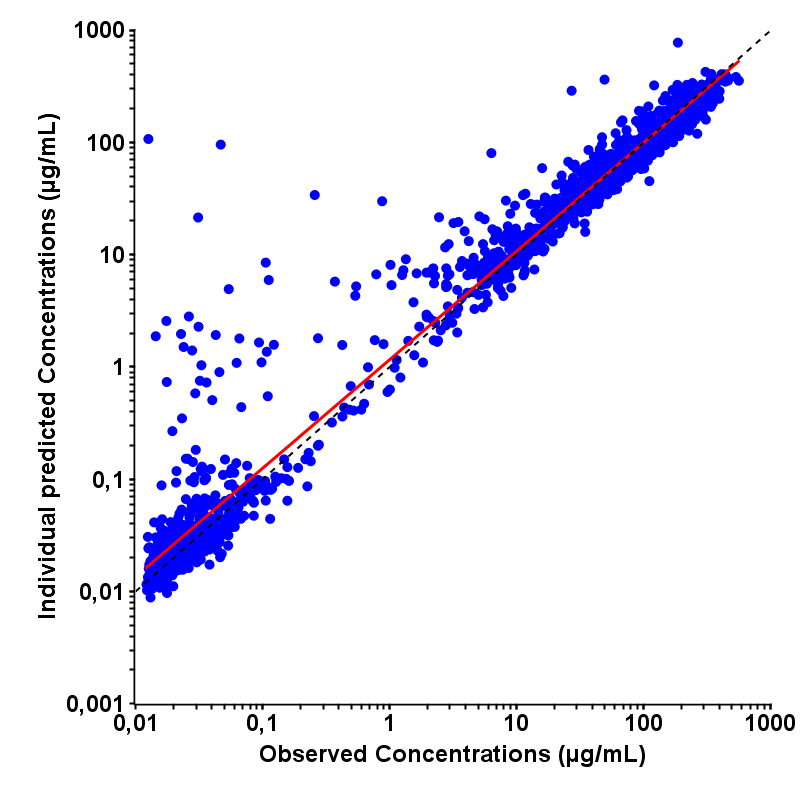 |
| IOPD patients (n=16) | |
| 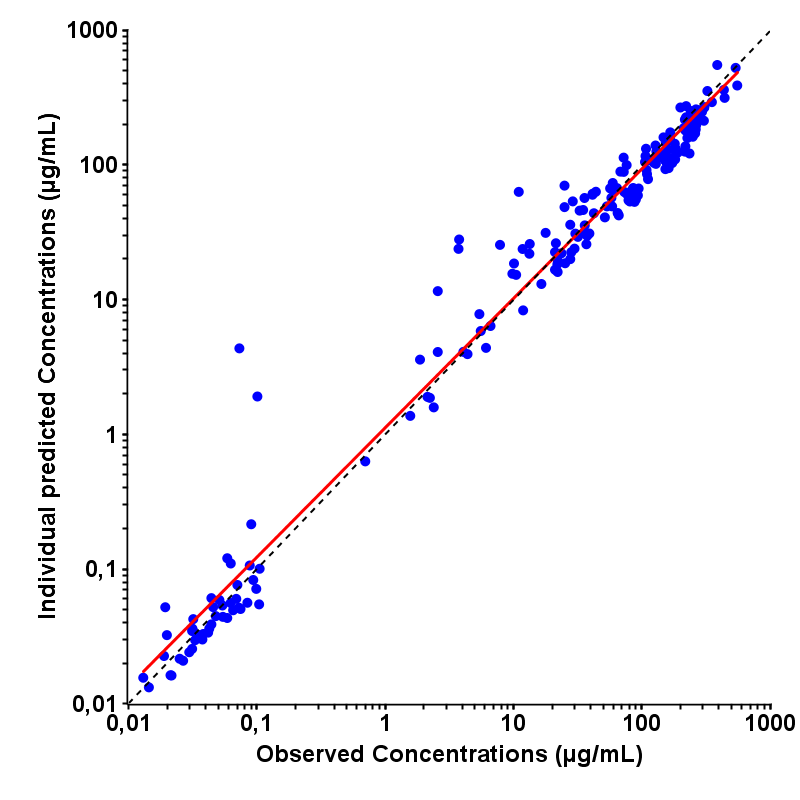 | 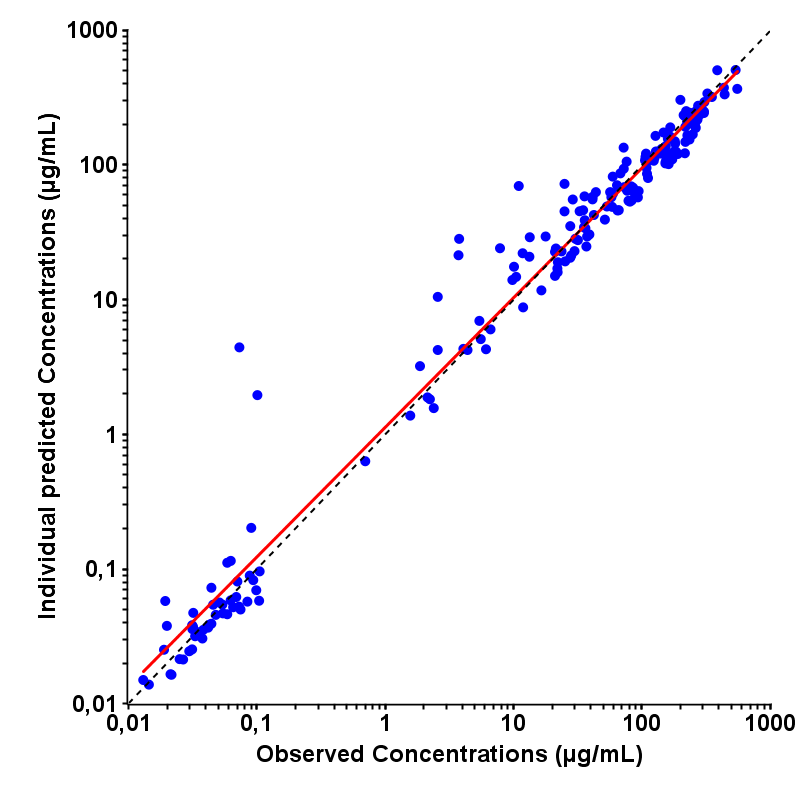 |

CWRES versus time after dose before (left) and after (right) covariate inclusion

| **Legend: Dashed black line: zero line – Red line: regression line** | |
| --- | --- |
| LOPD patients (n=75) | |
| 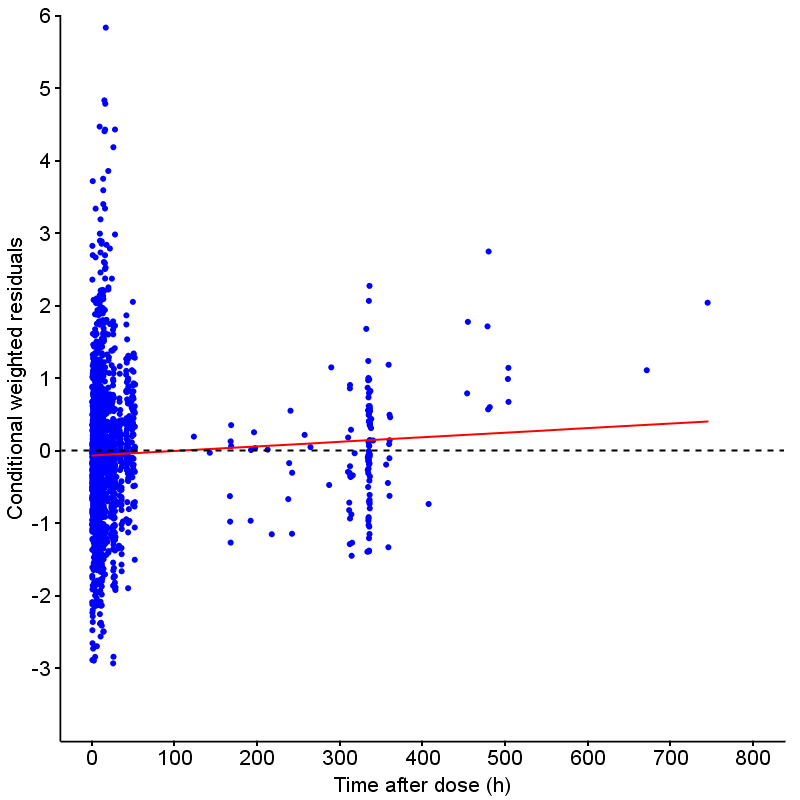 | 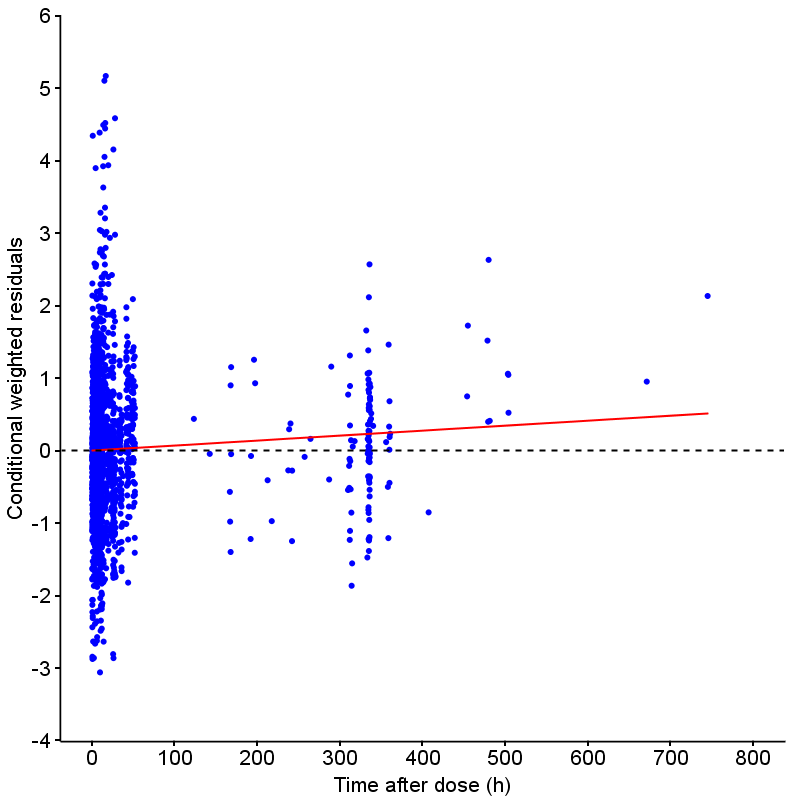 |
| IOPD patients (n=16) | |
| 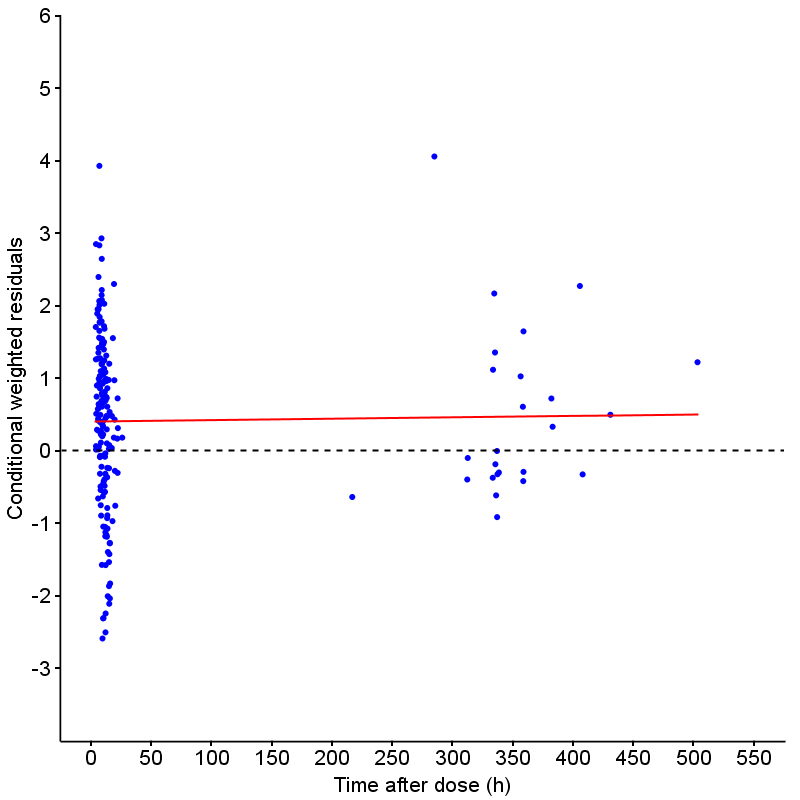 | 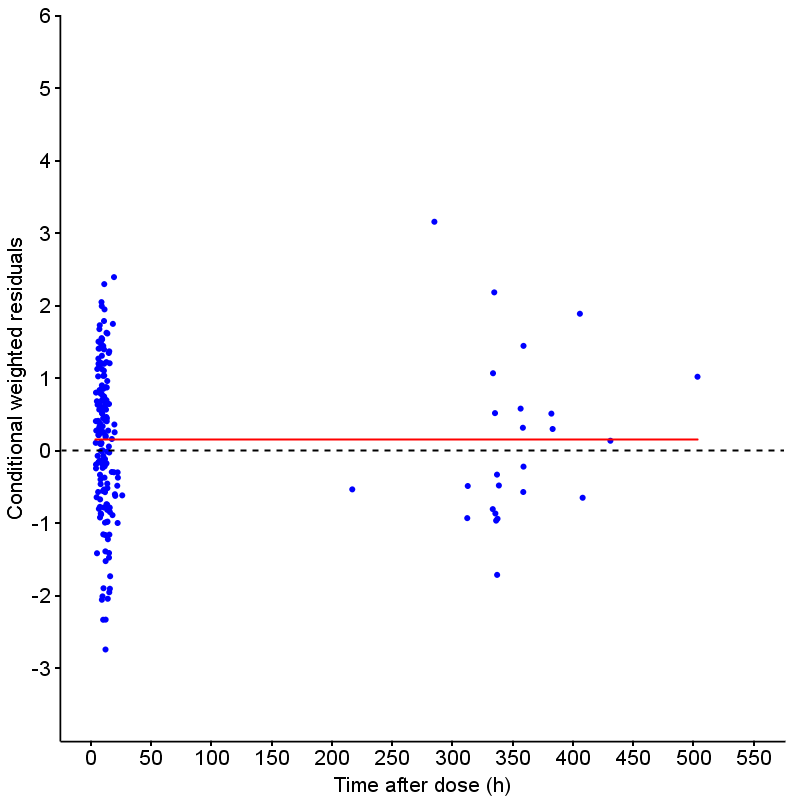 |

CWRES versus PRED before (left) and after (right) covariate inclusion

| **Legend: Dashed black line: zero line – Red line: regression line** | |
| --- | --- |
| LOPD patients (n=75) | |
| 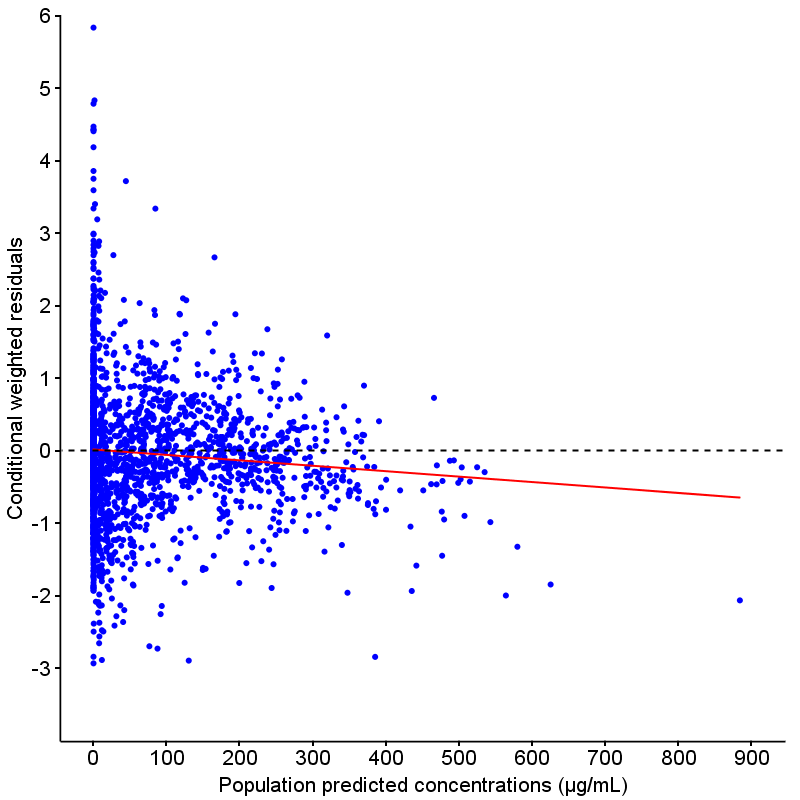 | 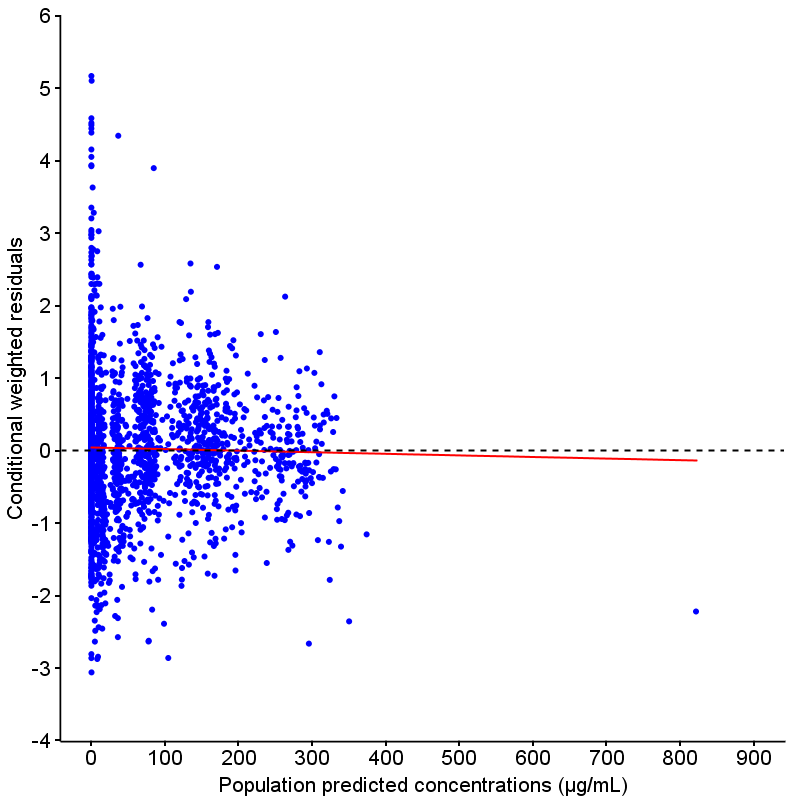 |
| IOPD patients (n=16) | |
| 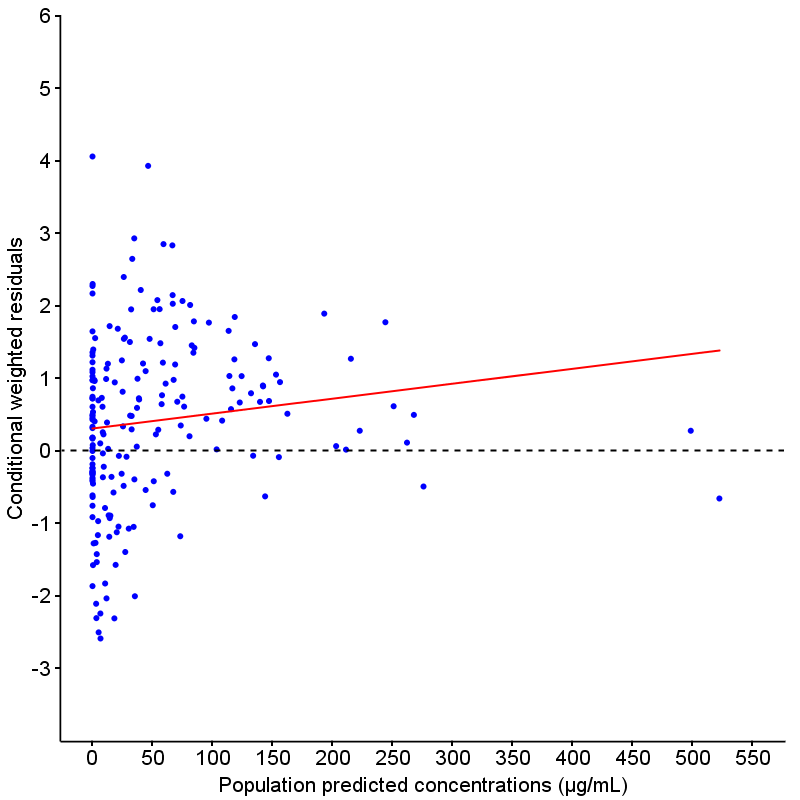 | 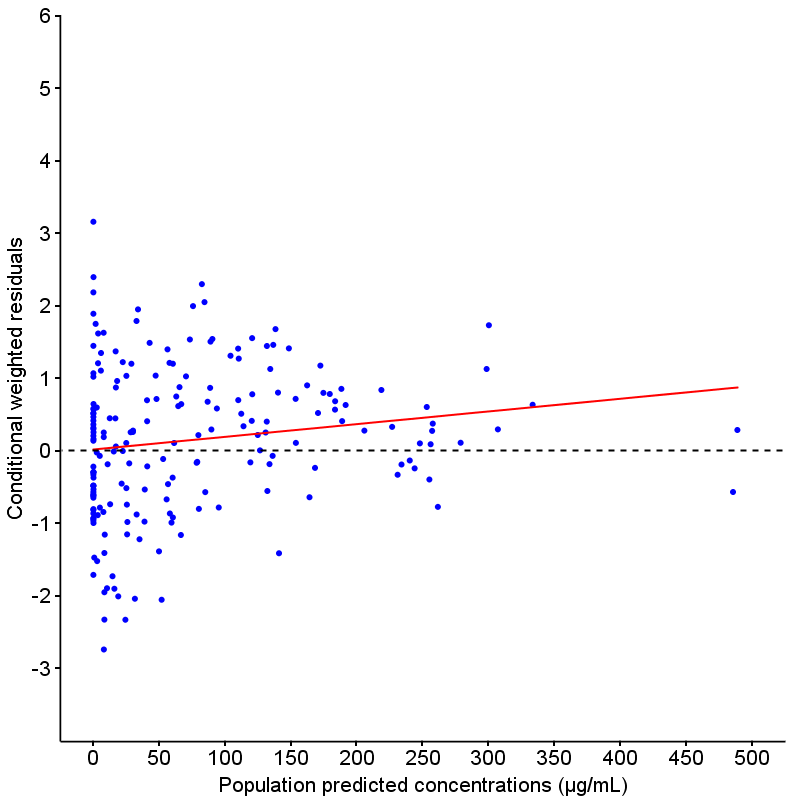 |

**Table 1: Summary of key selected runs for allometric scaling of parameters of the pharmacostatistical model**

| **NAME** | **Fixed allometric factor *a*** | **Optimized allometric**  **factor** | **validated job(s) *b*** | **Best OFV of validated job *b*** |
| --- | --- | --- | --- | --- |
| **PSM (no allometric scaling)** | **NA** | **NA** | **YES** | **5509** |
| CLV1V2V3_fixed | CL, V1,V2,V3 | NO | NO | NA |
| CL_V1_V2_V3 | NO | CL, V1, V2 and V3 | NO | NA |
| CL_Vm_V1_V2_V3 | NO | CL, V1, V2,V3 and Vm | NO | NA |
| **CL_V1_Vm *c*** | **NO** | **CL, V1 and Vm** | **YES** | **5295** |
| CL_Vm_V1V2V3_fixed | V1, V2,V3 | CL and Vm | NO | NA |
| Vm_CLV1V2V3_fixed | CL, V1,V2,V3 | Vm | NO | NA |
| WT_CL_Vm_V1_QPC | NO | CL, Vm, V1,QPC | NO | NA |
| CL_Vm_V1V2V3_com | NO | CL,Vm and common V1,V2,V3 | YES | 5317 |
| CL_Vm_QPC_V1V2V3_com | NO | CL,Vm,QPC and common V1,V2,V3 | YES | 5307 |
| CL_Vm_Q2_V1V2V3_com | NO | CL,Vm,Q2 and common V1,V2,V3 | NO | NA |
| CL_Vm_V1_V2V3_com | NO | CL,Vm,V1 and V2,V3 common | NO | NA |
| CL_Vm_V1_Q2_V2 | No | CL,Vm,V1,Q2 and V2 | NO | NA |
| OFV: Objective function value; NA: Not Applicable   1. Allometric scaling factor were kept fixed to a value of 0.75 for clearance and 1 for distribution volumes 2. All the tested models were extensively searched for successful result through an initial value parameter screening process: for each model 25 set of initial parameter values were randomly generated and optimized. A run was considered successful if minimization and covariance evaluation steps were completed successfully, all RSE were ≤50%, all correlation between parameters were ≤95%, the number of significant digit was ≥ 3.0 and the ratio between the highest and lowest eigen values of the correlation matrix of estimates was lower than 1000. 3. The selected allometric factor combination | | | | |

**Table 2: Quality criteria of the final model**

**Analysis of total clearance according to drug concentration and bodyweight of patients**

The computed total clearance versus AVAL concentrations for different body weights shows the non-linear toward linear behavior by increasing AVAL circulating concentration as well as the increasing effect of the bodyweight.

**Analysis of the apparent bodyweight normalized clearance according to the age of patients**

The individual computed apparent bodyweight normalized clearance highlight the decreasing effect of age for pediatric patients.

Legend: each patient of the dataset received a single dose of 20 mg/kg or 40 mg/kg and their corresponding AUC_2W_ evaluated. The apparent bodyweight normalized clearance was the computed as:

$$\frac{CL}{WT}=\frac{Dose}{AUC_{2W} X Body weight}$$
